# Oncological Surgical Wound Care: A Comparison of Theruptor NXT Non-adherent Dressing and the Current Standard of Care

**DOI:** 10.7759/cureus.56593

**Published:** 2024-03-20

**Authors:** Amritha Prabha Shankar, Kiran Kumar BR, Bharat Shankar, Ravoori H Babu, Rahul Dholariya, Priya, Shubhashree Muralidhar, Ganesh M Subramanya

**Affiliations:** 1 Surgical Oncology, Vydehi Institute of Medical Sciences and Research Centre, Bengaluru, IND; 2 Radiation Oncology, Vydehi Institute of Medical Sciences and Research Centre, Bengaluru, IND

**Keywords:** patient satisfaction, scar evaluation, theruptor nxt dressing, standard dressing, wound pain, surgical site infections

## Abstract

Background

Oncological surgeries pose an elevated risk of surgical site infections (SSIs) due to their complexity and various associated treatments, impacting patient outcomes and healthcare costs. This has prompted a focus on advanced wound dressings that provide microbial protection, exudate absorption, and improved product performance, enhancing patient satisfaction. Our study aimed to compare the efficacy and safety of Theruptor NXT with the current standard of care (SOC) practice involving cotton/povidone/micropore dressings in the postoperative wound management of oncological surgeries.

Methodology

A total of 102 patients who underwent oncological surgeries in the Department of Surgical Oncology, Vydehi Institute of Medical Sciences and Research Centre, Bengaluru, India between May and September 2023 were randomized to Theruptor NXT and SOC dressing groups (51 patients each). The incidence of SSIs, wound pain score, cosmetic appearance of the wound, and adverse events were assessed in the two groups at various intervals, i.e., post-surgery day 2 ± 1, day of discharge, and post-surgery day 30 ± 7. Further, the subject satisfaction and product usage were evaluated on post-surgery day 2 ± 1.

Results

The baseline characteristics were found to be comparable in both groups, i.e., Theruptor NXT and SOC groups. Further, the SSI rates, scar outcomes, and physiological parameters were also similar between the Theruptor NXT and SOC groups, indicating a similar safety profile of both dressings (p > 0.05). However, the product usage assessment revealed statistically significant differences, favoring Theruptor NXT in terms of superior ease of application, stretchability, exudate management, breathability, and non-adherence properties (p < 0.05).

Conclusions

Our findings suggest that Theruptor NXT wound dressing is a promising, effective, and user-friendly alternative to SOC wound dressing in diverse clinical settings.

## Introduction

Surgical site infections (SSIs) arising from invasive procedures constitute a significant proportion of healthcare-associated infections, accounting for 20% of such cases [1-3]. The US Centers for Disease Control and Prevention (CDC) reported an estimated 110,800 SSIs associated with inpatient surgeries in 2015, with a significant increase of 4% of the SSI standardized infection ratio [4]. However, their actual prevalence may be underestimated as they often occur after the discharge of the patient [5]. SSIs are associated with substantial morbidity with one-third of postoperative deaths linked to these infections [6]. The infection rates vary based on the type of surgical procedure, with “clean” surgeries exhibiting lower infection figures (3% to 5%) compared to procedures involving contaminated or necrotic tissues (10% to 30%) [7,8]. Wound infections typically present with erythema, discharge, and incision induration, occurring in 2-7% of patients around four to seven days post-surgery [9].

Globally, millions of surgical procedures are performed annually. In the context of the prevailing trend in cancer surgeries, the projected surge in the annual demand for cancer surgeries worldwide is anticipated to rise from 9 million procedures in 2018 to over 13.8 million surgeries by 2040 [10]. The majority of the surgeries involve wound closure using stitches, staples, clips, or glue for healing [7]. Dressings are commonly applied post-surgery to protect wounds from infection. Several trials have compared basic wound contact dressings such as gauze and surgical absorbents with film dressings such as Opsite (Smith & Nephew), Tegaderm (3M Healthcare), and Alginate (Medtronic) [11-13]. For instance, two different studies compared Trushield NXT non-adherent wound dressing with standard of care (SOC) dressing and Tegaderm, respectively, in postoperative wound management of obstetric and gynecological surgeries. The authors reported that Trushield NXT was superior to SOC dressing and comparable to Tegaderm [13,14]. The choice of wound dressing may impact surgical and patient outcomes such as SSI risk, scarring, pain reduction, patient acceptability, and ease of removal. Postoperative management involves the removal of dressing in a timely manner and addressing wound infections through various measures, including incision and drainage [7,13].

An advanced wound care product, Theruptor NXT non-adherent wound dressing, comprises a three-dimensional knitted hydrocellular textile substrate made of polyethylene terephthalate and polyurethane for optimal exudate management and moisture control (Healthium Medtech Ltd.). The dressing acts as a physical barrier against contaminants, promotes continuous infection control, and is non-adherent, non-leachable, and waterproof [15]. It contains permanently bound cationic sites that attract negatively charged pathogens, resulting in physical disruption in the cell wall of the pathogen. It has shown an efficacy of 4-log reduction against Gram-positive and Gram-negative bacteria, fungi, and yeast [15].

Many randomized trials have been reported in the literature that compared different types of dressing, such as silver, mupirocin, honey-based, vitamin E, and silicone-containing dressing, and negative pressure wound therapy in the wound management of cancer patients [16-19]. While various trials have investigated the effectiveness of different dressings in preventing SSIs in postoperative wound management of oncological surgeries, a significant gap persists in the existing body of evidence. This gap raises crucial questions about the optimal dressing choice and underscores the need for more comprehensive research to address this critical aspect of post-surgical care [20]. Therefore, this present study aimed to compare the efficacy and safety of Theruptor NXT with the current SOC practice involving cotton/povidone/micropore dressings in the postoperative wound management of oncological surgeries.

## Materials and methods

Trial design

This study was a single-center, prospective, two-arm, randomized design with a parallel group of 1:1. The study was designed to assess the safety and effectiveness of Theruptor NXT non-adherent wound dressing versus SOC dressing in postoperative wound management of oncological surgeries. It was conducted in the Department of Surgical Oncology, Vydehi Institute of Medical Sciences and Research Centre, Bengaluru, India. The study protocol was approved by the Institutional Ethics Committee of the hospital and registered with the Clinical Trial Registry of India (CTRI/2023/05/052574, registered on: 12/05/2023)). The study was performed following the principles defined in the Declaration of Helsinki and adhered to the 2010 Consolidated Standards of Reporting Trials (CONSORT) guideline for clinical trials [21].

Patients

All patients aged between 18 and 75 years who underwent uncomplicated oncological surgeries, had surgical incision size of ≥5 cm, had clean or contaminated surgical wounds, and willingly provided written informed consent were considered eligible for inclusion in the study.

Patients with systemic infection and/or local infection at the site of surgery, who had a life expectancy of fewer than six months, and those participating in another clinical trial fewer than 30 days before participation in the present trial were excluded from the study. In addition, the investigator exercised discretion in assessing individual cases based on clinical considerations to ensure a tailored approach for patient exclusion as needed.

Intervention

All surgical procedures were performed by attending surgeons. After surgery, wounds were cleaned and patients received their allocated wound care, i.e., Theruptor NXT or SOC wound dressing.

In the intervention group, the Theruptor NXT non-adherent wound dressing was applied on the wound bed and kept in place for two days. Conversely, standard care of dressing, i.e., povidone, cotton, and micropore was used in the control group and kept in place for two days.

Outcomes

The primary outcome of this study was to evaluate the incidence of SSI using the CDC criteria [4,22] on post-surgery day 2 ± 1, day of discharge, and post-surgery day 30 ± 7. The secondary outcome measured wound pain, patient satisfaction, cosmetic appearance of the wound using a Modified Hollander Wound Score, surgeon’s rating on product usage, and adverse events. The wound pain score and cosmetic appearance of the wound were assessed on post-surgery day 2 ± 1, day of discharge, and post-surgery day 30 ± 7 while subject satisfaction was evaluated on post-surgery day 2 ± 1 only and product usage on surgery day and post-surgery day 2 ± 1. The adverse events were assessed at all time intervals, i.e., surgery day, post-surgery day 2 ± 1, day of discharge, and post-surgery day 30 ± 7.

Data collection

On the screening day, comprehensive details were recorded encompassing demographic data (age, gender, and ethnicity), physical characteristics (height, weight, and body mass index (BMI)), medical history, and vital signs (temperature, pulse rate, respiratory rate, and blood pressure). On the day of surgery, vital signs and surgery-specific details such as type of surgery, length of incision, type of suture, and nature of intervention were documented. After the intervention, surgeons rated the product using the product usage assessment scale on surgery and post-surgery days and reported any adverse events encountered, if any. Thereafter, the surgeons assessed the wound and scored a Modified Hollander Wound Score on post-surgery day 2 ± 1, day of discharge, and post-surgery day 30 ± 7. Vital signs, SSI as per the CDC criteria, and adverse events were assessed on all visits after surgery. In addition, the patients were asked to rate patient satisfaction with wound dressing (the comfort of dressing while in usage and while removal on a five-point scale) on post-surgery day 2 ± 1 while wound pain score (10-point scale on the visual analog scale) on post-surgery day 2 ± 1, day of discharge, and post-surgery day 30 ± 7 [13]. The number of analgesics was also monitored throughout the study visits.

Sample size calculation

Following the guidelines outlined in Laura Flight and Steven. A. Julious’s Practical Guide to Sample Size Calculation [23], the sample size for this superiority trial was determined using key parameters as follows:

𝑧1−𝛼⁄2 (Z-value for 10% level of significance) = 1.645 for α = 0.05

𝑧1−𝛽 (Z-value for 80% power) = 0.84 for β = 0.2, σ = 19, when r = 1

A sample size of 92 (46 in each group) was deemed sufficient to detect a 10% difference in the proportion of patients with improved wound healing between groups at an 80% power and a 10% significance level. Anticipating a 10% dropout rate, the total number of patients enrolled in the study was determined to be 102 (51 in each group).

Randomization and blinding

A computer-generated randomization list with an allocation ratio of 1:1 was employed for intervention and assignment of dressing for patients. Surgeons maintained blindness to the treatment allocation throughout the surgery, revealing it only at the moment of applying the dressing. However, blinding could not be sustained during the follow-up period. This randomization process ensured unbiased evaluation of outcomes and enhanced the reliability of the results.

Statistical methods

The variations in the baseline characteristics and outcome measures among the study groups were compared using statistical tests, viz. chi-square test for nominal variables and the independent two-tailed Student’s t-test as parametric approach or Mann-Whitney U test as non-parametric approach for continuous variables. The statistical significance was set at a p-value <0.05. The statistical analysis was conducted using SPSS version 24 (IBM Corp., Armonk, NY, USA).

## Results

**Figure 1:**
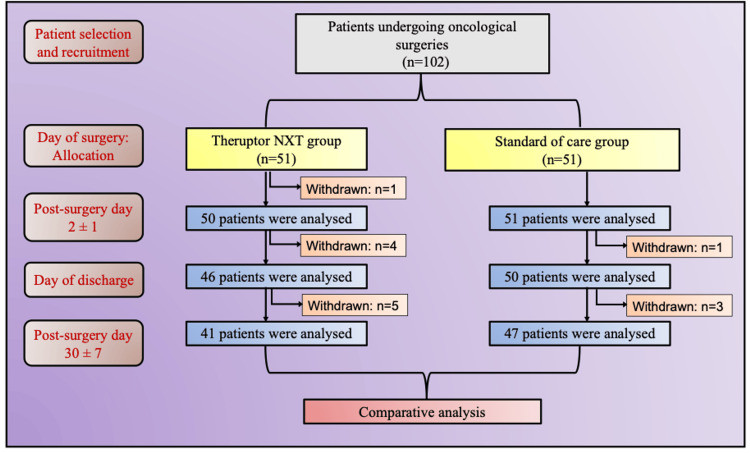
Consolidated Standards of Reporting Trials (CONSORT) flow diagram.

A total of 102 patients underwent oncological surgeries in the Department of Surgical Oncology, Vydehi Institute of Medical Sciences and Research Centre, Bengaluru, India between May and September 2023. The follow-up was completed in October 2023. In total, 14 patients were withdrawn during different periods of the study, as shown in Figure 1.

Baseline characteristics

The baseline characteristics and surgery details of the enrolled patients in the Theruptor NXT and SOC groups were compared and are shown in Table 1. No significant differences were observed between the groups in terms of age, weight, height, BMI, ethnicity, medical history, and blood transfusions (p > 0.05). The patients were diagnosed with malignancies of various sites, namely, the oral cavity (buccal mucosa and tongue), breast, stomach, thyroid, parotid gland, ovary, testis, lymphoma, colon, and rectum. Further, the type of surgery, length of incision, suture used, and type of suturing also showed no significant differences between the groups (p > 0.05).

**Table 1:** Baseline characteristics and surgery details of the enrolled patients. SOC = standard of care; HTN = hypertension; DM = diabetes mellitus; n = number of patients; SD = standard deviation

Characteristics (mean ± SD)	Theruptor NXT group (n = 51)	SOC group (n = 51)	P-value
Age (years)	49.71 ± 12.09	49.33 ± 12.90	0.927
Weight (kg)	62.42 ± 12.25	62.11 ± 17.43	0.936
Height (cm)	159 ± 9.28	157.06 ± 9.07	0.847
BMI (kg/m^2^)	24.77 ± 4.58	24.49 ± 4.66	0.848
Ethnicity, n (%)			0.937
Asian Indian	49 (96.1)	50 (98)	
Asian non-Indian	02 (3.9)	01 (2)	
Medical history, n (%)			0.747
HTN	12 (23.6)	09 (17.6)	
DM	13 (25.5)	10 (19.6)	
Thyroid	01 (2)	01 (2)	
Heart disease	02 (3.9)	01 (2)	
Details of surgery			
Type of surgery, n (%)			0.173
Mastectomy	14 (27.5)	09 (17.6)	
Wide local excision	07 (13.7)	05 (9.8)	
Midline laparotomy	12 (23.5)	18 (35.3)	
Transverse laparotomy	01 (2)	01 (2)	
Composite resection (buccal mucosa)	04 (7.8)	06 (11.8)	
Neck dissection	02 (3.9)	01 (2)	
Parotidectomy	05 (9.8)	00 (0)	
Thyroidectomy (hemi/total)	04 (7.8)	08 (15.7)	
Axillary dissection	02 (3.9)	03 (5.9)	
Length of incision (cm), mean ± SD	18.04 ± 7.72	19.13 ± 8.51	0.737
Suture used, n (%)			0.846
2-0 Ethilon polyamide	00 (0)	02 (3.9)	
2-0 Trulon polyamide	13 (25.5)	19 (37.3)	
2-0 Truglyde polyglycolic acid	03 (5.8)	01 (2)	
3-0 Trulon polyamide	12 (23.6)	09 (17.6)	
3-0 Monocryl poliglecaprone-25	01 (2)	02 (3.9)	
3-0 Truglyde polyglycolic acid	01 (2)	00 (0)	
3-0 Monoglyde poliglecaprone-25	21 (41.1)	18 (35.3)	
Type of suturing, n (%)			0.757
Continuous	22 (43.1)	21 (41.1)	
Interrupted	29 (56.9)	30 (58.9)	
Blood transfusions, n (%)	07 (13.7)	08 (15.7)	0.848

In addition, the vital signs remained comparable throughout the study period, as shown in Table 2.

**Table 2:** Vitals of the study participants. SOC = standard of care; n = number of patients; SD = standard deviation

Parameters (mean ± SD)	Theruptor NXT group	SOC group	P-value
Screening day	n = 51	n = 51	
Temperature (°F)	97.54 ± 1.013	97.65 ± 0.94	0.947
Respiratory rate (beats/minute)	17.25 ± 2.39	17.1 ± 2.33	0.837
Pulse rate (beats/minute)	84.29 ± 12.94	85.67 ± 13.16	0.526
Systolic blood pressure (mmHg)	116.27 ± 12.37	119.52 ± 15.22	0.242
Diastolic blood pressure (mmHg)	74.82 ± 10.25	75.29 ± 9.41	0.638
Surgery day	n = 51	n = 51	
Temperature (°F)	97.75 ± 0.66	97.73 ± 0.81	0.948
Respiratory rate (beats/minute)	17.05 ± 1.89	17.29 ± 1.88	0.826
Pulse rate (beats/minute)	84.31 ± 10.68	85.07 ± 10.15	0.546
Systolic blood pressure (mmHg)	115.96 ± 11.75	119.25 ± 14.18	0.236
Diastolic blood pressure (mmHg)	75.88 ± 8.02	76.35 ± 9.03	0.628
Post-surgery day 2 ± 1	n = 50	n = 51	
Temperature (°F)	97.65 ± 0.8	97.7 ± 0.87	0.945
Respiratory rate (beats/minute)	16.62 ± 1.57	16.96 ± 2.01	0.847
Pulse rate (beats/minute)	83.9 ± 10.27	86.15 ± 11.61	0.529
Systolic blood pressure (mmHg)	115.49 ± 12.54	118.54 ± 16.99	0.239
Diastolic blood pressure (mmHg)	75.33 ± 7.93	75.76 ± 8.86	0.621
Day of discharge	n = 46	n = 50	
Temperature (°F)	97.62 ± 0.62	97.74 ± 0.75	0.992
Respiratory rate (beats/minute)	16.52 ± 1.54	16.62 ± 1.63	0.847
Pulse rate (beats/minute)	83.34 ± 9.7	84.2 ± 7.97	0.524
Systolic blood pressure (mmHg)	116.61 ± 12.71	118.2 ± 13.37	0.246
Diastolic blood pressure (mmHg)	75. 69 ± 7.75	77.04 ± 8.25	0.695
Post-surgery day 30±7	n = 41	n = 47	
Temperature (°F)	97.64 ± 0.52	97.4 ± 0.94	0.928
Respiratory rate (beats/minute)	16.19 ± 1.3	16.03 ± 1.47	0.836
Pulse rate (beats/minute)	81.55 ± 8.77	84.9 ± 5.83	0.548
Systolic blood pressure (mmHg)	116.06 ± 12.76	119.54 ± 12.58	0.229
Diastolic blood pressure (mmHg)	75.09 ± 6.61	76.25 ± 7.01	0.642

SSI assessment

The assessment of SSI was conducted at several time points, i.e., post-surgery day 2 ± 1, day of discharge, and post-surgery day 30 ± 7. Both the Theruptor NXT and SOC groups revealed no statistically significant difference in the incidence of SSIs (p > 0.05) throughout the study. The majority of patients in both groups remained free from infections during follow-up visits, as shown in Table 3. Moreover, no adverse events were reported by any patient at any time during the study.

**Table 3:** SSI assessment. SSI = surgical site infections; SOC = standard of care; n = number of patients

SSI, n (%)	Theruptor NXT group	SOC group (n = 51)	P-value
Post-surgery day 2 ± 1	n = 51	n = 51	
Yes	00 (0)	01 (2.2)	0.855
No	51 (100)	50 (97.8)	
Day of discharge	n = 46	n = 50	
Yes	02 (4.4)	03 (6)	0.899
No	44 (95.6)	47 (94)	
Post-surgery day 30 ± 7	n = 41	n = 47	
Yes	01 (2.5)	01 (2.2)	0.984
No	40 (97.5)	46 (97.8)	

Product usage assessment scale

On the surgery day, the surgeons were asked to rate the dressing product on a five-point scale, with 1 denoting poor and 5 excellent. The findings indicated statistically significant differences between the Theruptor NXT and the SOC groups, favoring the Theruptor NXT group being excellent in terms of ease of application (28% vs. 5.9%), stretchability/flexibility (26% vs. 11.8%), and time taken for application of dressing (30.33 ± 14.39 vs. 37.67 ± 19.01) (p < 0.05). Further, the properties of dressing such as exudate management (20% vs. 4%), breathability of skin (16% vs. 2%), conformance to skin (18% vs. 0%), stickiness of adhesive layer (18% vs. 2%), waterproofing property (14% vs. 2%), ease of removal (10% vs. 2%), and non-adherent properties (16% vs. 4%) were compared on post-surgery day 2 ± 1, and Theruptor NXT wound dressing was found to be superior compared to the SOC dressing (p < 0.05) (Table 4).

**Table 4:** Product usage assessment scale. SOC = standard of care; n = number of patients

Product usage assessment scale, n (%)	Theruptor NXT group	SOC group	P-value
Surgery day	n = 51	n = 51	
Ease of application			0.011
Fair	04 (8)	01 (2)	
Good	11 (22)	21 (41.1)	
Very good	22 (44)	26 (51)	
Excellent	14 (28)	03 (5.9)	
Stretchability/Flexibility			0.048
Poor	00 (0)	01 (2)	
Fair	04 (8)	05 (9.8)	
Good	34 (66)	39 (76.4)	
Very good	13 (26)	06 (11.8)	
Time taken for application of dressing (seconds), mean ± SD	30.33 ± 14.39	37.67 ± 19.01	0.031
Post-surgery day 2 ± 1	n = 50	n = 50	
Exudate management			0.031
Fair	01 (2)	01 (2)	
Good	13 (26)	27 (54)	
Very good	26 (52)	20 (40)	
Excellent	10 (20)	02 (4)	
Breathability of skin			0.003
Fair	01 (2)	00 (0)	
Good	07 (14)	29 (58)	
Very good	34 (68)	20 (40)	
Excellent	08 (16)	01 (2)	
Conformance to skin			0.001
Fair	05 (10)	04 (8)	
Good	13 (26)	40 (80)	
Very good	23 (46)	06 (12)	
Excellent	09 (18)	00 (0)	
Stickiness of the adhesive layer			0.04
Fair	01 (2)	03 (6)	
Good	15 (30)	39 (78)	
Very good	25 (50)	07 (14)	
Excellent	09 (18)	01 (2)	
Waterproofing property			0.015
Poor	00 (0)	01 (2)	
Fair	00 (0)	07 (14)	
Good	15 (30)	26 (52)	
Very good	28 (56)	15 (30)	
Excellent	07 (14)	01 (2)	
Ease of removal			0.022
Fair	00 (0)	08 (16)	
Good	13 (26)	26 (52)	
Very good	32 (64)	15 (30)	
Excellent	05 (10)	01 (2)	
Non-adherent			0.008
Fair	00 (0)	04 (8)	
Good	08 (16)	24 (48)	
Very good	34 (68)	20 (40)	
Excellent	08 (16)	02 (4)	

Patient satisfaction

Patients belonging to the Theruptor NXT group rated the product better for comfortable usage (28% vs. 10%) and removal (8% vs. 2%) than the SOC group and the difference was statistically significant (p < 0.05). The Theruptor NXT group reported higher proportions of patients with very good and excellent ratings for both comfortable usage and removal, as shown in Table 5. Concerning wound pain and the number of analgesics used, no significant differences were observed between the Theruptor NXT and SOC groups across all follow-up time points (p > 0.05).

**Table 5:** Patient satisfaction on product usage and wound pain. SOC = standard of care; SD = standard deviation; n = number of patients

Parameters, n (%)	Theruptor NXT group	SOC group	P-value
Surgery day	n = 50	n = 50	
Comfortable usage			0.014
Fair	00 (0)	01 (2)	
Good	06 (12)	20 (40)	
Very good	30 (60)	24 (48)	
Excellent	14 (28)	05 (10)	
Comfortable removal			0.002
Fair	00 (0)	02 (4)	
Good	13 (26)	32 (64)	
Very good	33 (66)	15 (30)	
Excellent	04 (8)	01 (2)	
Pain during dressing removal			0.062
Low	43 (86)	35 (70)	
Moderate	7 (14)	15 (30)	
Wound pain, mean ± SD	2.64 ± 0.72	2.49 ± 0.61	0.742
Number of analgesics used, mean ± SD	1.4 ± 0.53	1.39 ± 0.53	0.876
Day of discharge	n = 46	n = 50	
Wound pain, mean ± SD	2.02 ± 0.68	2.1 ± 0.91	0.843
Number of analgesics used, mean ± SD	1.17 ± 0.53	1.26 ± 0.44	0.597
Post-surgery day 30 ± 7	n = 41	n = 47	
Wound pain, mean ± SD	0.85 ± 0.52	0.81 ± 0.64	0.653
Number of analgesics used, mean ± SD	0.37 ± 0.58	0.21 ± 0.46	0.19

Modified Hollander Wound Score

The scar evaluation was conducted using the Modified Hollander Wound Score Scale among two groups, i.e., Theruptor NXT and SOC groups. The Modified Hollander Wound Score Scale comprises six parameters, namely, step-off borders, contour irregularities, margin separation, edge inversion, excessive distortion, and overall appearance, which were scored as 0 and 1 for the absence and presence of these features, respectively. Based on the assessment parameters, no statistically significant differences were observed between the groups on the post-surgery day 2 ± 1 (p > 0.05). Similar trends were observed on the day of discharge and post-surgery day 30 ± 7, revealing no significant differences in scar evaluation parameters between the groups (p > 0.05), as shown in Table 6.

**Table 6:** Scar evaluation among the groups. SOC = standard of care; 0 = feature absent; 1 = feature present; n = number of patients

Modified Hollander Wound score scale, n (%)	Theruptor NXT group	SOC group	P-value
Post-surgery day 2 ± 1	n = 50	n = 50	
Step-off borders			0.942
0	50 (100)	49 (98)	
1	00 (0)	01 (2)	
Contour irregularities			0.837
0	49 (98)	50 (100)	
1	01 (2)	00 (0)	
Margin separation			0.942
0	50 (100)	49 (98)	
1	00 (0)	01 (2)	
Edge inversion			1.000
0	50 (100)	50 (100)	
1	00 (0)	00 (0)	
Excessive distortion			0.837
0	49 (98)	50 (100)	
1	01 (2)	00 (0)	
Overall appearance			0.723
0	47 (94)	50 (100)	
1	03 (6)	00 (0)	
Total score			0.623
0	46 (92)	48 (96)	
1	03 (6)	02 (4)	
2	01 (2)	00 (0)	
Day of discharge	n = 46	n = 50	
Step-off borders			0.738
0	45 (97.8)	48 (96)	
1	01 (2.2)	02 (4)	
Contour irregularities			0.861
0	44 (95.6)	49 (98)	
1	02 (4.4)	01 (2)	
Margin separation			0.748
0	42 (91.2)	48 (96)	
1	04 (8.8)	02 (4)	
Edge inversion			0.837
0	45 (97.8)	49 (98)	
1	01 (2.2)	01 (2)	
Excessive distortion			0.738
0	45 (97.8)	48 (96)	
1	01 (2.2)	02 (4)	
Overall appearance			0.738
0	45 (97.8)	48 (96)	
1	01 (2.2)	02 (4)	
Total score			0.763
0	41 (89)	48 (96)	
1	02 (4.4)	00 (0)	
2	02 (4.4)	00 (0)	
4	01 (2.2)	00 (0)	
5	00 (0)	02 (4)	
Post-surgery day 30 ± 7	n = 41	n = 47	
Step-off borders			0.963
0	40 (97.6)	46 (97.9)	
1	01 (2.4)	01 (2.1)	
Contour irregularities			0.912
0	39 (95.1)	46 (97.9)	
1	02 (4.9)	01 (2.1)	
Margin separation			0.963
0	40 (97.6)	46 (97.9)	
1	01 (2.4)	01 (2.1)	
Edge inversion			0.836
0	40 (97.6)	47 (100)	
1	01 (2.4)	00 (0)	
Excessive distortion			1.000
0	41 (100)	47 (100)	
1	00 (0)	00 (0)	
Overall appearance			0.963
0	40 (97.6)	46 (97.9)	
1	01 (2.4)	01 (2.1)	
Total score			0.738
0	38 (92.8)	45 (95.7)	
1	01 (2.4)	00 (0)	
2	01 (2.4)	02 (4.3)	
4	01 (2.4)	00 (0)	

## Discussion

Our study assessed the efficacy and safety of Theruptor NXT wound dressing compared to SOC in post-surgical wound management of oncological surgeries by covering several aspects, including SSI assessment, physiological parameters, pain score, scar evaluation, patient satisfaction, product usage assessment, and adverse events. Based on the results of this study, we found that Theruptor NXT performed better than SOC in post-surgical wound management in terms of product usage characteristics and patient satisfaction, thereby making it a potential option for the advancement of oncological post-surgical wound care.

In this study, the baseline characteristics, medical history, surgical details, and vital signs of the patients belonging to both groups, i.e., Theruptor NXT and SOC groups were comparable. These findings suggest that the two groups were well-matched, thus strengthening the validity of the comparative analysis in subsequent assessments [24,25]. Notably, our study did not emphasize on a particular cancer but covered a broad range of oncological surgeries.

Further, the assessment of SSI showed no significant differences between the Theruptor NXT and the SOC groups at several time points of the study, i.e., post-surgery day 2 ± 1, day of discharge, and post-surgery day 30 ± 7. Both groups exhibited comparable rates of SSI, indicating that both Theruptor NXT and SOC dressings do not pose an increased risk of infections. The findings ensure patient safety and align with the primary goal of wound management, which is to promote healing while minimizing complications [7,26]. In a study, Biffi et al. (2012) assessed and compared the SSI rate in patients with colorectal cancer elective surgery using silver and common dressing. The authors found no significant differences in the SSI rate between the groups, suggesting equal efficacy of both dressings [27], which is similar to our findings. Moreover, Gupta et al. (2022) conducted an in vitro study on the antimicrobial properties of Theruptor and found that Theruptor dressing significantly reduced the growth of microorganisms, suggesting the efficacy of dressing against a broad spectrum of pathogens [21].

Modern wound dressings can maintain temperature, are non-adhesive to tissues, maintain a moist environment, and aid in pain relief to promote wound healing [28]. In our study, the product usage assessments provided insights into the surgeons’ experience with Theruptor NXT compared to the standard dressing in which surgeons rated Theruptor NXT superior in terms of ease of application, stretchability/flexibility, and a faster application process (p < 0.05). Similar to Theruptor NXT, Trushield NXT non-adherent wound dressing is also formulated on DTAC technology [13,14]. In a randomized study, Ray et al. (2022) compared Trushield NXT with SOC dressing and found Trushield NXT better than SOC in terms of exudate management, breathability of skin, conformance to skin, waterproofing property, ease of removal, and non-adherent properties [13]. In accordance with this study, our results revealed the advantages of Theruptor NXT in exudate management, breathability of skin, conformance to skin, waterproofing property, ease of removal, and non-adherent properties during the follow-up period. The statistically significant differences favor Theruptor NXT, highlighting its user-friendly attributes and potential to enhance patient comfort during the post-surgical period. These results also support the suitability of Theruptor NXT as an effective alternative to the standard dressing in post-surgery wound management. Similar to our study, Bredow et al. (2018) compared Mepilex-Border dressing with conventional dressing in the postoperative management of orthopedic surgery and reported intervention dressing was better than conventional dressing in terms of patient satisfaction and surgeon ratings. However, no difference was observed in postoperative complications [29].

In a review, desJardins-Park et al. (2019) mentioned that most wounds heal by scarring, resulting in devastated functional and aesthetic wounds [30]. In our study, the scar evaluation by the Modified Hollander Scale revealed comparable outcomes between groups, including step-off borders, contour irregularities, margin separation, edge inversion, excessive distortion, overall appearance, and total score. Our findings suggest that both wound dressings do not compromise the aesthetic aspects of wound healing and scar formation.

Strengths and limitations

The study demonstrates several strengths that contribute to its scientific validity and applicability. First, the randomized controlled trial design employed in the research enhances the internal validity of the findings, as it helps control for potential confounding variables and ensures a more reliable comparison between Theruptor NXT and the SOC dressing groups. The inclusion of 102 patients from a specific department specializing in surgical oncology adds credibility to the results, as it increases the generalizability of the findings to the target population. Despite these strengths, the relatively small sample size and single-center study design, focusing only on patients with oncological surgeries might limit the generalizability of the findings to broader clinical settings and diverse patient populations. Additionally, the short follow-up duration of the study may restrict the ability to capture long-term outcomes or delayed complications associated with the wound dressings. Another limitation of the study is the inclusion of patients with clean wounds. Further studies with patients having dirty wounds will provide comprehensive results. Lastly, 14 patients were withdrawn during the study due to their index condition.

## Conclusions

The results of this study suggest that Theruptor NXT wound dressing is a potential and advantageous alternative to the standard dressing in post-surgical wound management of oncological surgeries. Theruptor NXT was found to be superior in product usage characteristics and patient satisfaction. Further studies with larger sample sizes and diverse patient populations could provide additional insights and strengthen the evidence supporting the adoption of Theruptor NXT in clinical practice.
